# Microplasma Jet Arrays as a Therapeutic Choice for Fungal Keratitis

**DOI:** 10.1038/s41598-018-20854-8

**Published:** 2018-02-05

**Authors:** Hyun Jung Park, Soon Hee Kim, Hyung Woo Ju, Hyesook Lee, Yoonjin Lee, Sehyun Park, Heejun Yang, Sung-Jin Park, J. Gary Eden, Jaewook Yang, Chan Hum Park

**Affiliations:** 1 Nano-Bio Regenerative Medical Institute, Hallym University, Chuncheon, Gangwon Republic of Korea; 2 T2B Infrastructure Center for Ocular Diseases, Inje University Busan Paik Hospital, 75 Bokji-ro, Busanjin-gu Busan, Republic of Korea; 3 Laboratory for Optical Physics and Engineering, Department of Electrical and Computer Engineering, University of Illinois, Urbana, IL USA; 4 Department of Ophthalmology, Inje University College of Medicine, 75 Bokji-ro, Busanjin-gu Busan, Republic of Korea; 5 Department of Otorhinolaryngology–Head and Neck Surgery, Chuncheon Sacred Heart Hospital, School of Medicine, Hallym University, Chuncheon, Gangwon Republic of Korea

## Abstract

The clinical impact of microplasma jets on rabbit eyes infected by *Candida albicans* has been investigated. Arrays of such jets produce low-temperature plasma micro-columns suitable for ophthalmic therapeutics and fungal infections, in particular, and the technology is capable of being scaled to surface areas of at least 10 cm^2^. Keratitis was induced in the right central corneas of rabbits, whereas the left eyes served as a normal group. The rabbits were divided into the plasma non-treated group (control) and plasma treatment group. Histologic analyses of both groups showed marked reductions in the thickness, angiogenesis, and opacity of all rabbit corneas following plasma treatment. Indeed, for treatment times beyond 14 days, infected eyes exhibited no significant differences from the normal group. Healing of rabbit eyes infected by *Candida albicans* apparently proceeds by disrupting corneal epithelial proliferation, and by reducing fibrotic changes in the stroma. This study demonstrates that low-temperature plasma jets are remarkably effective in healing *Candida albicans*-infected corneas, thereby providing a promising medical treatment option for keratitis.

## Introduction

Mycotic keratitis exists in two forms, the first of which is caused by filamentary fungi (*Fusarium* and *Aspergillus*) whereas the second results from yeast-like fungi such as *Candida albicans*. While the former is generally related to a single predisposing factor, keratitis produced by the yeast-like fungus can develop as a result of multiple factors such as an ocular defect (including insufficient tear secretion and defective eyelid closure) or systemic conditions such as immunosuppression or diabetes mellitus. The yeast *Candida albicans* is the most common cause of keratitis and endophthalmitis^1,2^. Another common source of corneal infection is the contact lens. Consequently, although keratitis resulting from yeast-like fungi has been more prevalent in temperate climates in the past, its reach now extends worldwide^3–6^.

Antifungal agents are known to be effective in the treatment of mycotic keratitis^3,7–11^. Topical amphotericin B is often prescribed for treating keratitis for yeast-like fungi, and topical natamycin is generally the choice for keratitis resulting from filamentary fungi^12^. Unfortunately, both require high dosages because ocular penetration is low, and side-effects are problematic. It is not surprising, therefore, that a significant fraction of patients (15–27% of those with fungal keratitis) require one or more surgical procedures, including debridement, corneal transplantation, or a bandage contact lens^13–16^.

The application of gas phase plasma to medicine and medical therapeutics, specifically, has accelerated in the last decade. The effectiveness of plasma tools in cutting, cauterizing, drying or coagulating tissue or blood^17^ is well-known but the gas temperatures characteristic of conventional thermal plasmas have deleterious effects on tissue in electrosurgery, for example. Desiccation of cells, protein denaturation, and the devitalization of tissues in such instances are common^18^. The recent emergence of microplasmas - low-temperature plasmas produced in cavities of mesoscopic dimensions - has transformed the therapeutics landscape because gas temperatures within the plasma are typically below 400–450 K, thus eliminating concern for damaging tissue^18,19^. Not surprisingly, therefore, microplasmas and other “cold” plasma technologies have quickly made inroads into dermatology, oncology, otolaryngology, gastroenterology, and odontology^20,21^. As one example, we recently reported the treatment of second-degree burns *in vitro* and *in vivo* with arrays of microplasma jets^20,22^.

The introduction of atmospheric pressure plasma technology into ophthalmology has lagged that of other medical disciplines. Specifically, few studies of mycotic keratitis have been conducted although the antimicrobial impact of plasma on various ocular pathogens has been reported^19,20^. It has been demonstrated that ocular pathogens such as *Candida albicans* and *Pseudomonas aeruginosa* are deactivated completely by 30–120 seconds of plasma treatment *in vitro*. Thus, plasma has been shown to be an effective ocular disinfectant for bacteria or fungi-contaminated cells or *ex vivo* human corneas.

We report here the clinical effectiveness of low-temperature plasma for treating *Candida albicans in vivo* with a rabbit model. In particular, the therapeutic effect of an array of microplasma jets on an ophthalmic condition has been investigated which demands an unusual degree of caution so as to avoid cellular damage. Microplasma arrays developed at the University of Illinois offer the spatial uniformity and area coverage that are essential to treating ocular infections quickly and effectively^21,23^. The primary function of each microplasma jet in an array has been shown to be the generation of atomic and molecular species which diffuse to the surface of the tissue and deactivate pathogens. Packing densities of plasma jets as high as 121 in a cross-sectional area of a few square cm have been reported^20,24,25^. Here, we introduce a dome-shaped polymer shroud (cap) to the microplasma jet array for the purpose of creating above the tissue to be treated an artificial chemical environment, one not confined solely to the atomic and molecular species available with plasmas formed from room air. By sealing the region of tissue to be treated, we are able to control the gas phase chemistry and, therefore, the identity of the molecular radicals (such as excited states of OH, O_2_ and N_2_) that impinge upon and interact with the tissue.

## Results

### Comparison of a single plasma jet device with microplasma jet arrays

**Figure 1:**
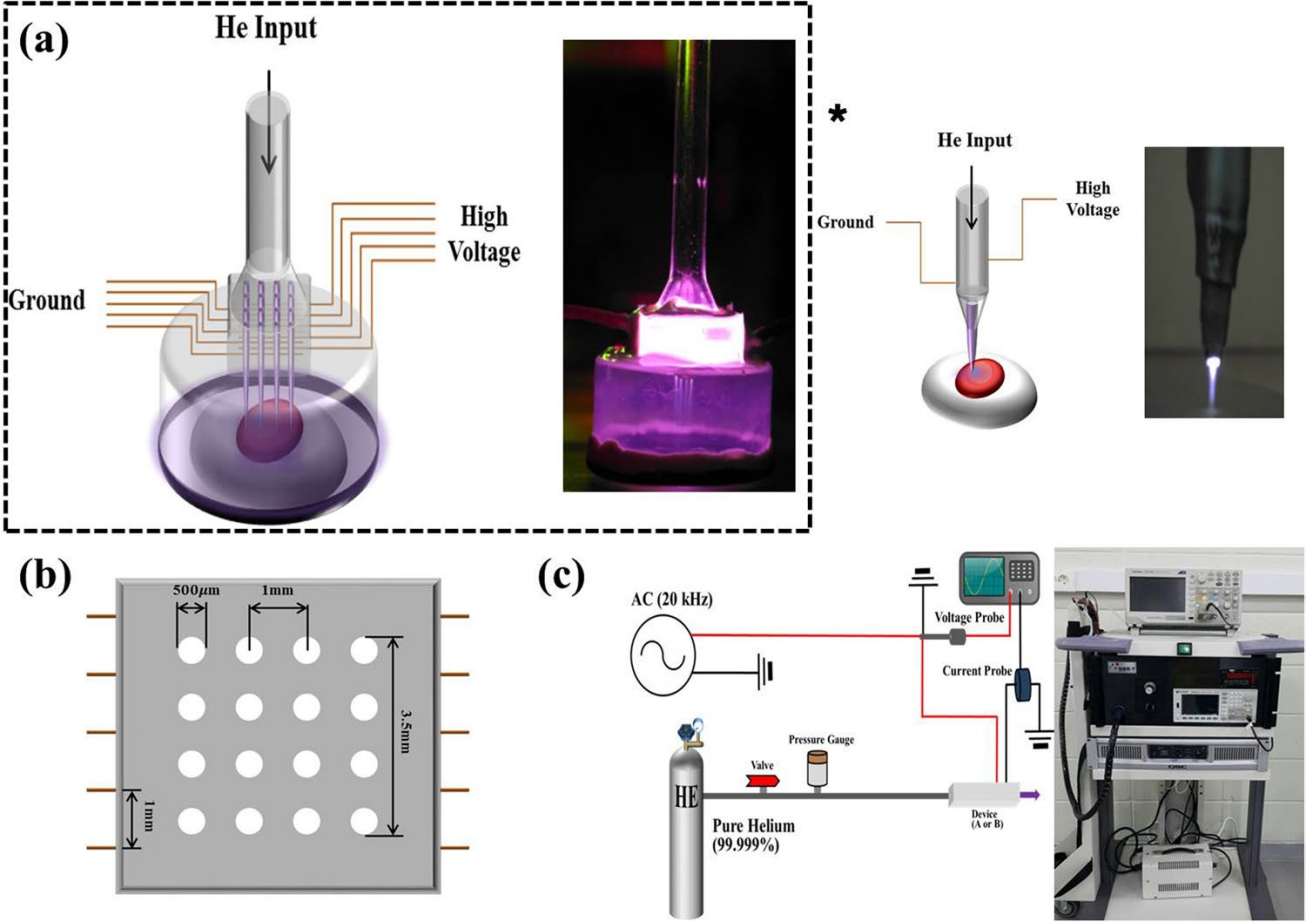
Schematic diagram of the structure of a cold atmospheric plasma jet. (**a**) General view of devices of microplasma jet (4 × 4 array) and single jet (asterisk: 1 × 1 array) with an electrical configuration (left) and photograph of perspective view operating with He input pressure of 780 Torr (right), (**b**) the front view of 4 × 4 microplasma jet and (**c**) schematic of the experimental set-up.

**Figure 2:**
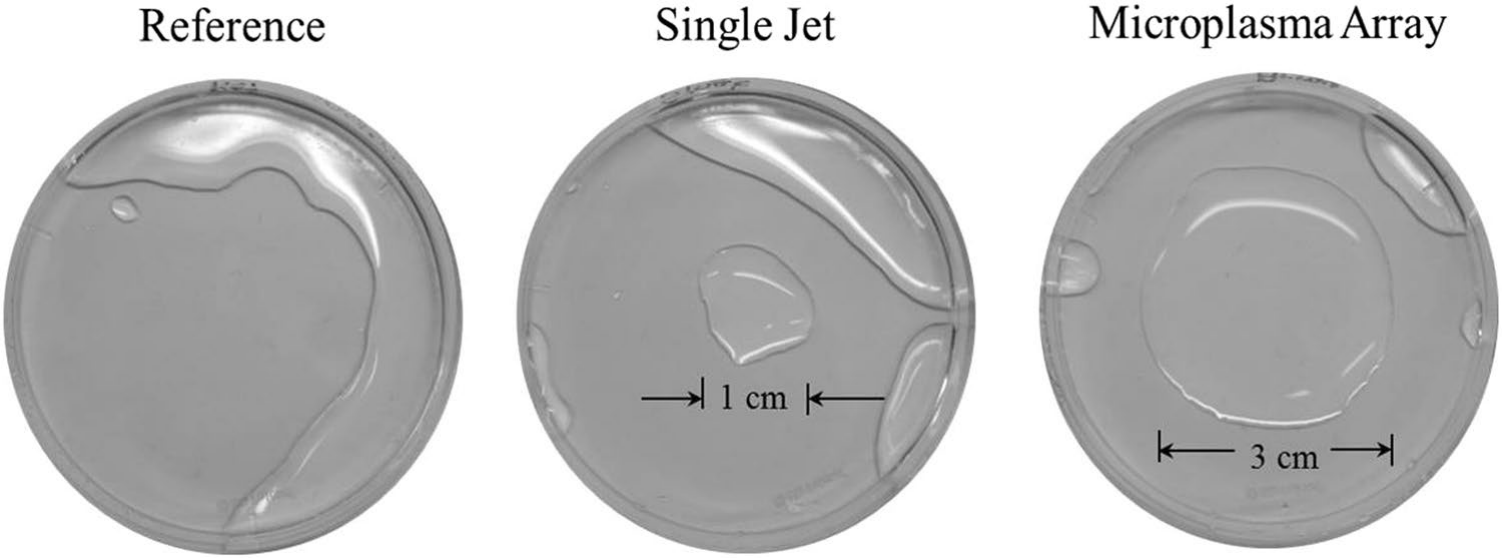
Photographs showing hydrophilicity of water at the surface treated area by plasma jet devices. The treatment was made for 3 seconds with each device. (left) control; (center) single plasma jet device (right) microplasma jet array with a cap enclosure (diameter of 3.5 cm).

**Figure 3:**
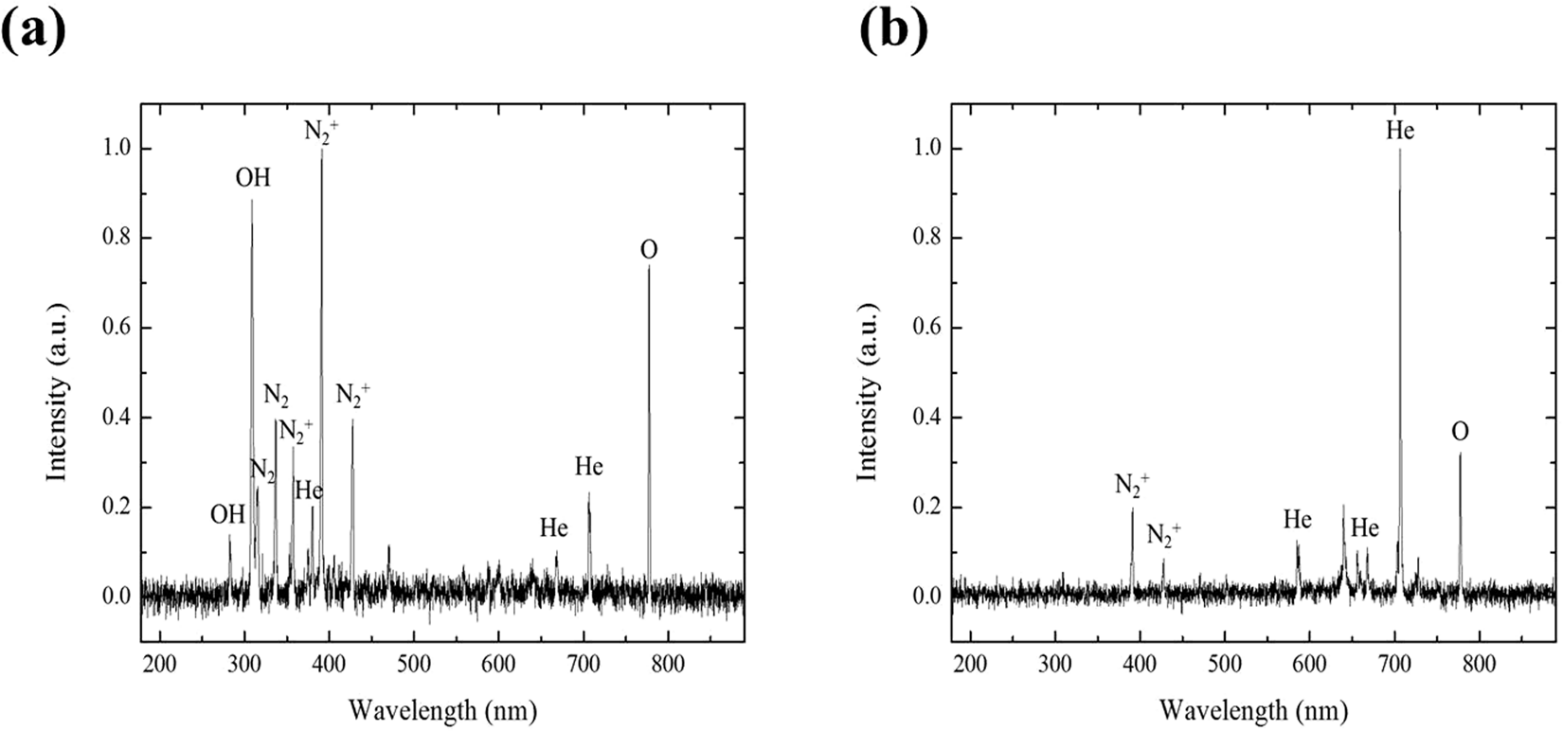
Optical emission spectra of cold plasma jet devices measured from end-on orientation to the jets. (**a**) Single plasma jet device having 2 mm diameter (**b**) 4 × 4 microplasma jet array.

Fig. 1 shows plan-view diagrams of two representative type of plasma jet devices fabricated at the University of Illinois. Fig. 2 shows photographs illustrating the dramatic changes occurring to a hydrophilic (polypropylene) surface as a result of its exposure to plasma jets. After a 3 s treatment with a single plasma jet, the treated area exhibited surface modification over an area 1 cm in diameter that corresponds to the region over which the incoming plasma and gas flow diffused on the surface. In contrast, an identical surface treated by a microplasma jet array reveals a modified region that is spatially uniform over the array area but also extends beyond, uniformly covering the entire area of the 3 cm diameter shroud. As illustrated in Fig. 3, the emissive species in the plasma jet change significantly when the plasma is no longer in continuous contact with room air. Panel (a) of Fig. 3 is a spectrum in the 200–800 nm wavelength region, detailing the radiating species in a single plasma jet. The dominant spectral features are those of the N_2_^+^(B–X) transition, the A–X band of the hydroxyl radical (OH), and electronically excited nitrogen. However, when a microplasma jet array is covered with a hemispherical polymer dome such that the shroud contacts the tissue to be treated (Fig. 3(b)), the emission lines observed shift almost entirely to those of the feedstock gas, helium. However, radiation from the nitrogen dimer ion, N_2_^+^, is also present in Fig. 3(b).

This difference between the two spectra of Fig. 3 has, we believe, profound implications for medical therapeutics because the philosophy underlying tissue treatment has been altered significantly. When one or more tissue-treatment plasmas is in contact with air, much of the energy in the plasma is stored in the form of molecular radicals such as OH. It is these radicals that interact with the surface and effect the desired therapeutic benefit. The drawback of this approach is that the clinician is limited to the gases available in room air, and the radicals and excited species derived from such a mixture by a low-temperature plasma. While radicals such as NO and OH, in particular, may be desirable for therapeutic applications, air may not afford the ideal gas mixture from which to produce the radicals. Furthermore, the radicals formed in an atmospheric glow plasma may produce radicals in the ground state, as opposed to an electronic state having several eV of internal energy. With the cap in place, however, the gaseous and chemical environments lying immediately above the tissue can be tailored so as to promote the production of species having larger internal energies (divided between vibrational, rotational, and electronic energy). In the case of Fig. 3(b), most of the identifiable peaks are attributable to He and, therefore, to states lying more than 20 eV above ground. It is clear that the chemistry at the surface of the tissue will also be altered when such a plasma impinges on the tissue.

### Changes to corneal tissue following treatment with non-thermal plasma jets

**Figure 4:**
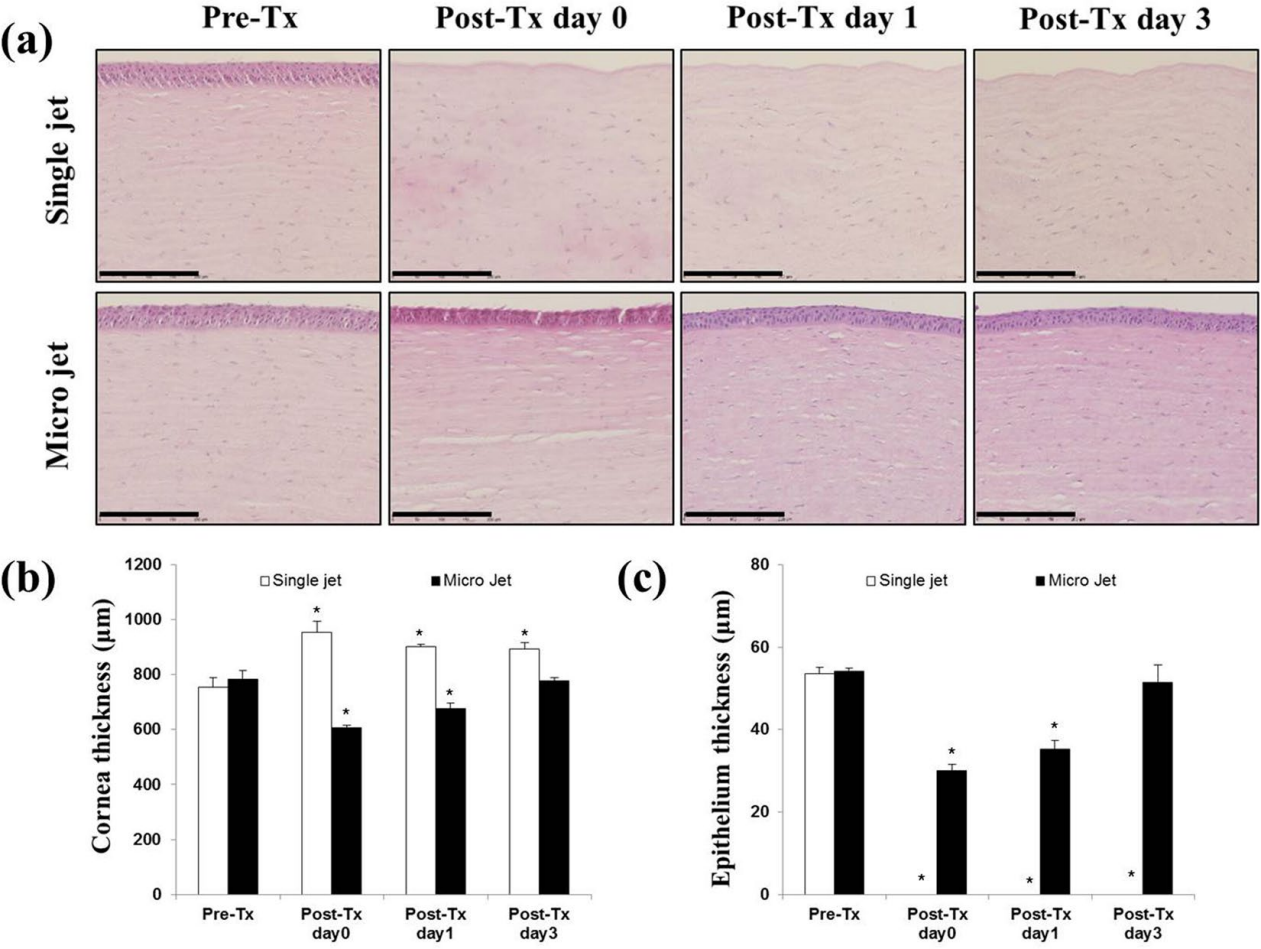
The effect of different type of plasma on the normal cornea. (**a**) The corneas after plasma treatment were stained with H&E. Images of the sections were photographed with a virtual microscope (NanoZoomer 2.0 RS, Hamamatsu, Japan). Scale bar, 200 μm. (**b**) Graph illustrating corneal thickness and (**c**) epithelial thickness. The data is shown as mean ± standard deviation (n = 4). Statistically significant by Tukey’s test at *p < 0.05 *vs* pre-Tx.

In order to observe the response of a rabbit cornea to treatment by different plasma sources, the left and right eyes of the rabbits were treated separately by the two types of plasma jets. The results were examined histologically, and the optical images of Fig. 4 illustrate the increase in corneal thickness, from 753.25 ± 32.17 µm (pre-treatment) to 954.00 ± 42.32 µm (post-treatment), that occurred in response to exposure of the tissue to a single plasma jet. In this case, corneal edema was induced, as evidenced by the 27% increase in corneal thickness following treatment. Furthermore, the corneal epithelial layer was lost entirely during treatment, falling from 53.70 ± 0.86 µm to zero (to within measurement uncertainty). In the case of the microplasma jet array, however, the overall corneal thickness decreased during treatment by only 28%, from 787.25 ± 29.64 µm to 603.75 ± 11.09 µm. Similarly, the epithelium thickness decreased during treatment by 44% (54.33 ± 1.95 µm pre-treatment, 30.25 ± 1.072 µm post treatment), thus demonstrating that the microplasma jet array reduces corneal edema to a greater extent than a single (larger diameter) jet, while partially preserving the corneal epithelium. In terms of the recovery, in the case of the single plasma jet, the cornea edema was slightly decreased (902.00 ± 9.64 μm) at 1 day after application, but not as a condition of pre-treatment. The epithelium was not also recovered on Day 3 after treatment. On the other hand, in the group of microplasma jet array, reduced the corneal thickness and epithelium thickness were recovered to the condition of the normal eye at Day 3 after treatment (777.67 ± 10.69 μm for cornea and 51.33 ± 4.13 μm for epithelium). Moreover, as shown in Supplementary data (Fig. S2), the application of microplasma jet showed no damages to surrounding tissues such as lens, iris, and retina. We conclude, therefore, that microplasma jets are preferable to a single plasma jet for the treatment of keratitis, and only microplasma jet arrays were used in the experiments described in the sections to follow.

### Effect of plasma treatment on corneal opacification and neovascularization in *Candida albicans*–infected rabbit models

**Figure 5:**
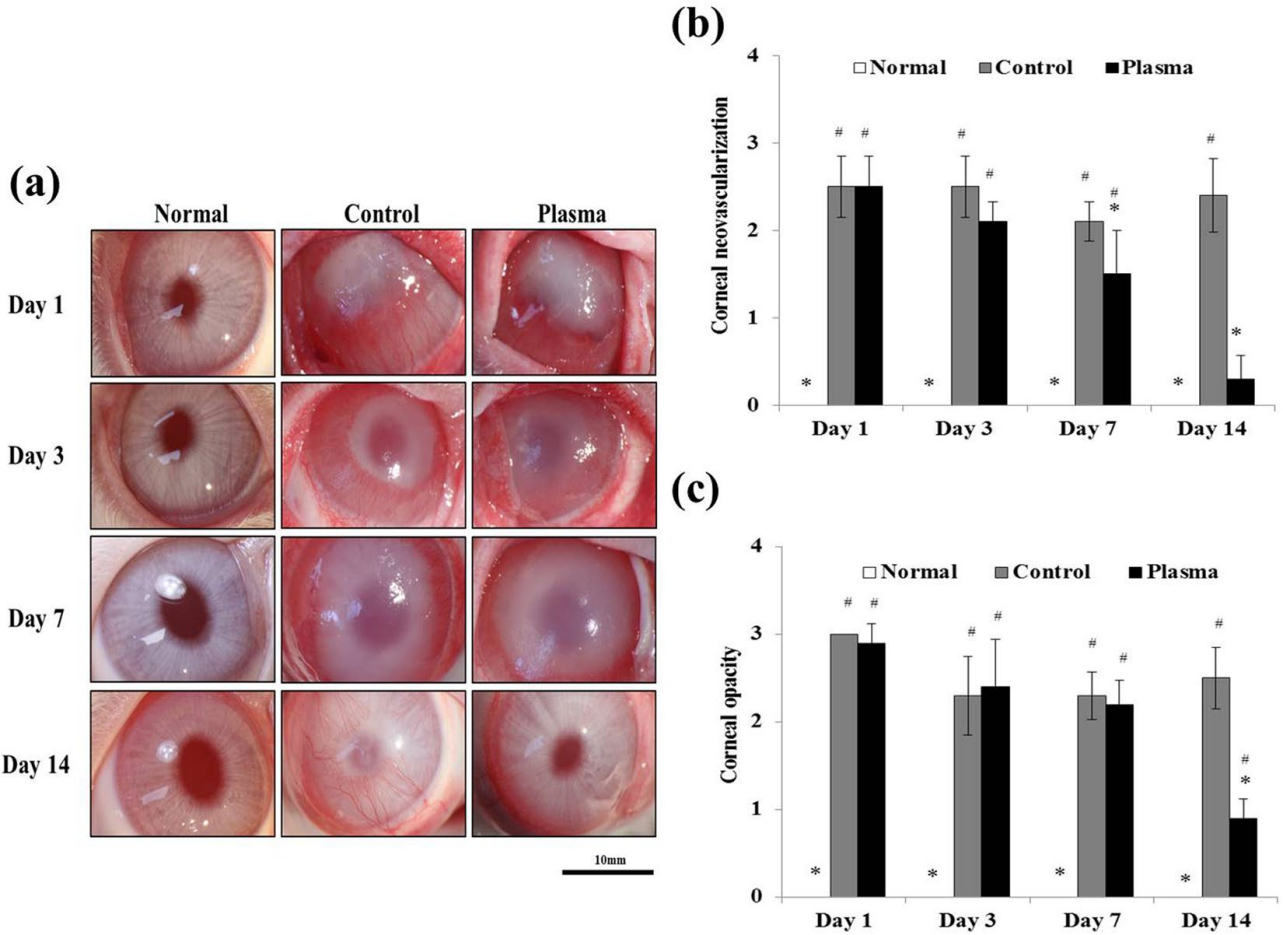
Effect of plasma on clinical outcomes in *Candida albicans*-infected rabbit. (**a**) Photographs of rabbits at 1, 3, 7 and 14 days after plasma treatment. The rabbits were randomly divided into two groups: the control group (plasma non-treatment), and plasma treatment group. Left eyes were used as a normal group. Images of the eye were obtained with a microscope (SZX7, Olympus, Tokyo, Japan). Scale bar, 10 mm. (**b**) Graphs presenting corneal neovascularization and (**c**) opacification. Data are expressed as the mean ± standard deviation (n = 4). Statistically significant by Tukey’s test at *p < 0.05 *vs* control and #*p* < 0.05 *vs* normal group.

The effectiveness of microplasma jet arrays in treating *Candida albicans* infections was evaluated on the basis of several corneal properties, foremost of which are neovascularization and corneal haze (examples are given in Fig. 5). Specifically, corneal fungal infections resulted in angiogenesis as well as an increase in the opacification of the rabbit cornea (cf. Fig. 5(a)). The level of corneal neovascularization in both the corneal limbus and central cornea was observed, as shown by the images in Panel (b) of Fig. 5. In the control group, the corneal limbus and central cornea maintained a relatively stable level of neovascularization throughout a 14 day period (i.e., 2.40 ± 0.42). However, in the case of the plasma-treated group, neovascularization was observed only in the central cornea until day five (2.10 ± 0.22). On day seven, angiogenesis also decreased (to 1.50 ± 0.50, *p* less than 0.05). After 14 days of plasma treatment, the infected corneas were restored to levels of angiogenesis characteristic of the normal (control) group (0.30 ± 0.27, *p* less than 0.05). It is concluded that corneal vascularization was diminished significantly by exposure of rabbit corneas to the array of microplasma jets.

Considering now the parameter of corneal opacification (Fig. 5(c)), the data show that opacity was maintained over time at a relatively constant level in the control group (2.50 ± 0.35). However, corneal opacification in the plasma-treated group decreased significantly in 14 days to 0.9 ± 0.22, demonstrating a level of improvement that rendered the infected eyes as indistinguishable from the control group with respect to opacification. These results demonstrate unambiguously that plasma treatment results in healing of corneal fungal infections, and should be considered as a treatment modality for keratitis.

### Effect of plasma treatment on histological changes of the cornea in *Candida albicans*–infected rabbit models

**Figure 6:**
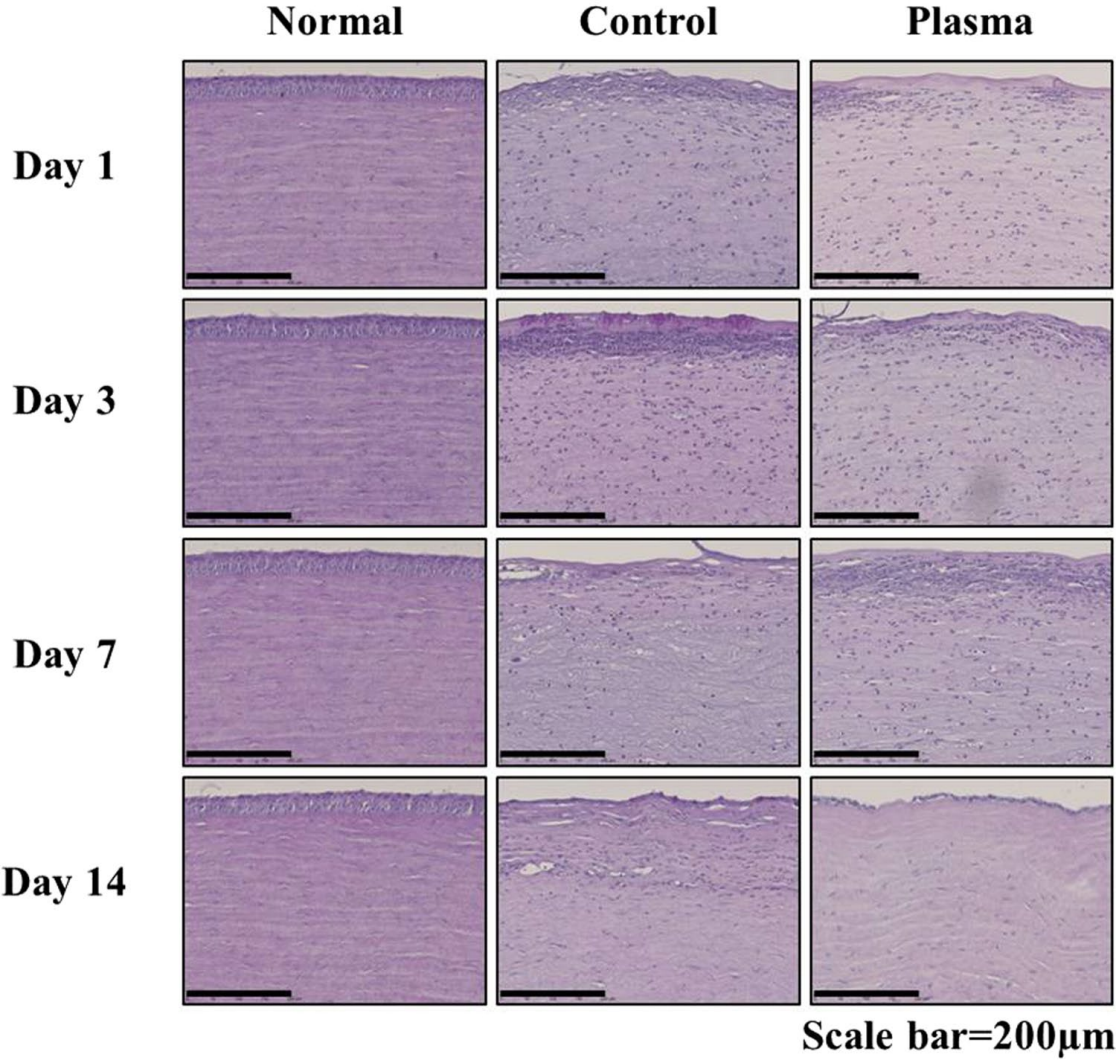
Effect of plasma on the histological change in *Candida albicans*-infected rabbit. The corneas were stained with PAS. Images of the sections were photographed with a virtual microscope (NanoZoomer 2.0 RS, Hamamatsu, Japan). Scale bar, 200 μm.

The distribution of *Candida*, and the histological changes, in the cornea following plasma treatment of *Candida albicans*- infected rabbit corneas are presented in the result of Periodic Acid-Shiff reagent (PAS) staining (Fig. 6**)**. In the control group, we observed significant damage to the corneal epithelium as well as stromal edema, as compared with normal tissue. Specifically, the *Candida* infection extended from the epithelial surface down into the deeper stromal layers. However, for the plasma-treated group, the *Candida* infection showed significant reduction inside the stroma. Also, plasma treatment resulted in the suppression of stromal swelling.

### Immunohistochemistry of angiogenesis markers

**Figure 7:**
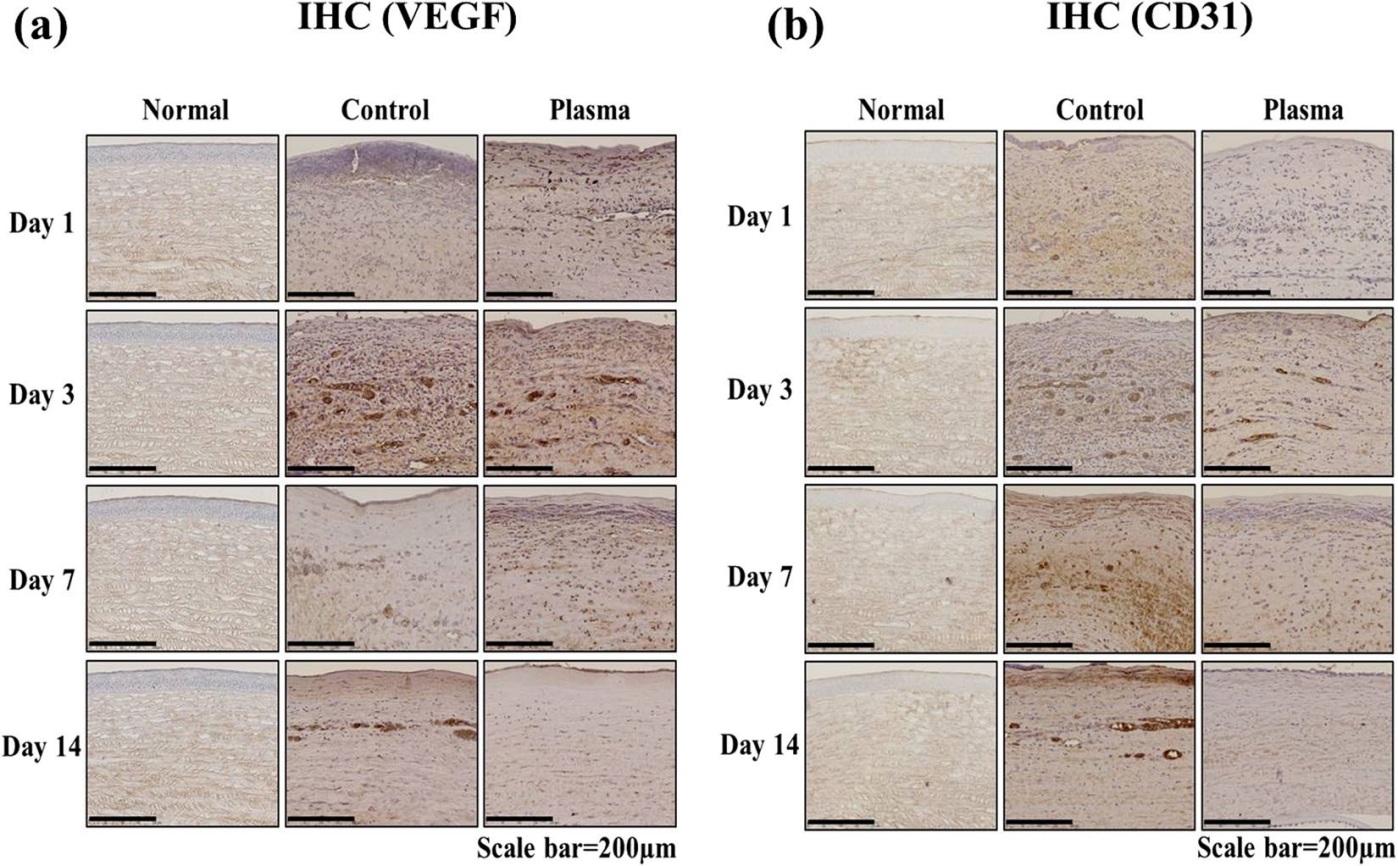
Effect of plasma on the expression of angiogenic marker VEGF and CD31 in *Candida albicans*-infected rabbit. The corneas were immunostained with specific antibodies for (**a**) VEGF and (**b**) CD31. The VEGF and CD31-expressed parts were shown in brown color. Images of the sections were photographed with a virtual microscope (NanoZoomer 2.0 RS, Hamamatsu, Japan). Scale bar, 200 μm.

As shown in Fig. 7, *Candida albicans* infections induce angiogenesis in rabbit corneas. Angiogenesis markers VEGF and CD31 were observed in corneal tissue through immunohistochemistry. In the control group, VEGF expression was induced to a significant degree in the inner layer of the substrate. In contrast, the corneal tissue of the plasma-treated group exhibited a monotonic reduction of VEGF expression with treatment time, as compared to the control group. On day 14, the level of VEGF expression had fallen to that for a normal (uninfected) eye. Furthermore, the results for the expression of CD31 were found to be quite similar to those for the VEGF marker. In the control group, the CD31 expression level increased with time, while plasma-treatment again suppressed the rate of expression. After 7 and 14 days of plasma treatment, rabbit corneas showed strong suppression of CD 31 expression which leads one to conclude: 1) *Candida albicans* infections induce neovascularization in corneal tissue, accompanied by VEGF and CD 31 marker expression, but 2) neovascularization of rabbit corneas and the expression of the VEGF and CD 31 angiogenesis markers are both strongly suppressed through the treatment of the corneal tissue with microplasma jets. These experiments suggest that low-temperature plasma microjets are an effective treatment against fungal *Candida* and should be investigated further as a medical treatment option for infective keratitis.

### Immunohistochemistry of inflammation markers

**Figure 8:**
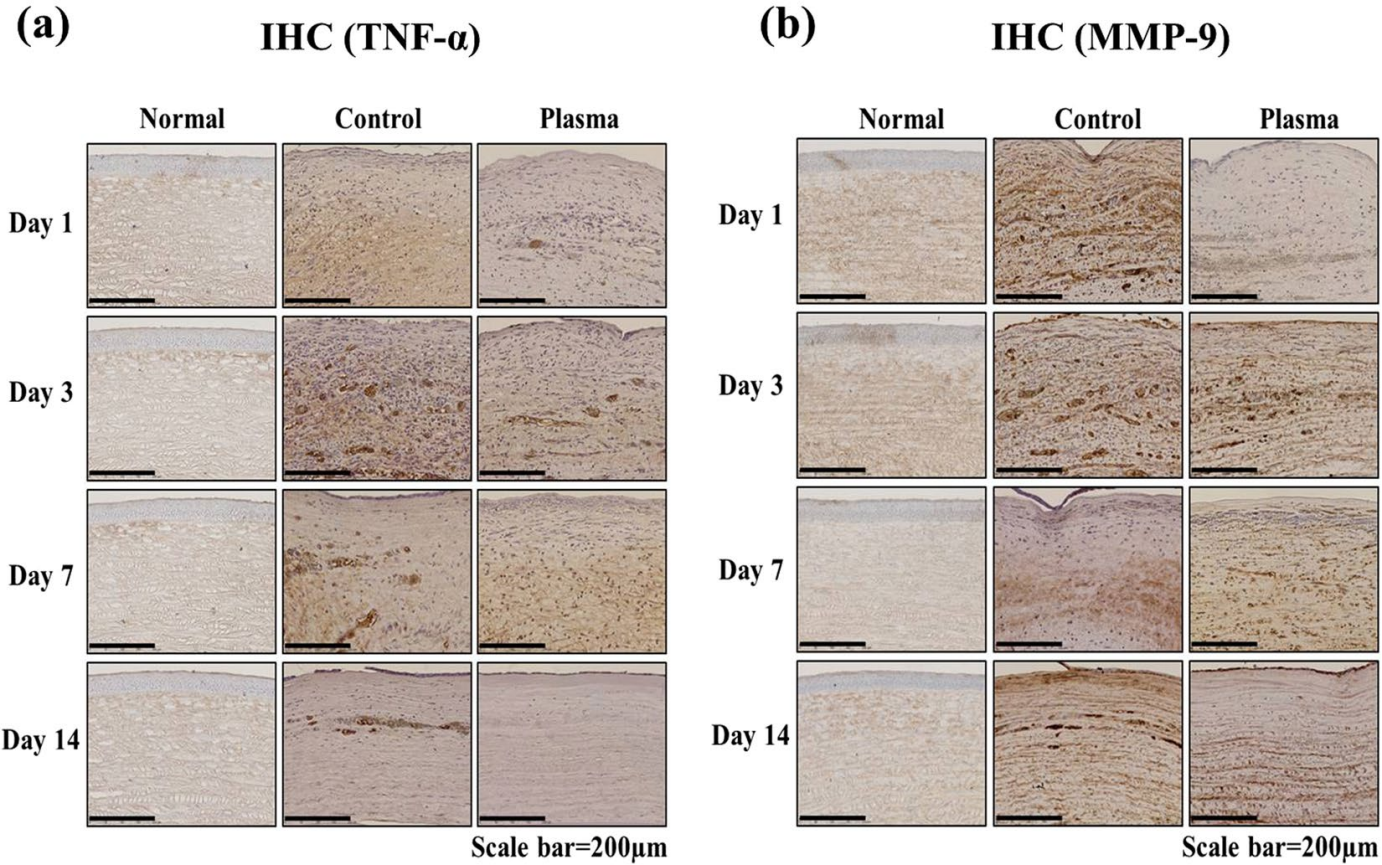
Effect of plasma on the expression of inflammatory marker TNF-α and MMP-9 in *Candida albicans*-infected rabbit. The corneas were immunostained with specific antibodies for (**a**) TNF-α and (**b**). The TNF-α and MMP-9-expressed parts were shown in brown color. Images of the sections were photographed with a virtual microscope (NanoZoomer 2.0 RS, Hamamatsu, Japan). Scale bar, 200 μm.

In an effort to evaluate the impact of the plasma jet treatment of *Candida albicans* infections, the inflammatory markers TNF-α and MMP-9 were also observed by immunohistochemistry **(**Fig. 8**)**. In these experiments, the corneas were immunostained with specific antibodies for TNF-α and MMP-9 and, in Fig. 8, the regions of tissue in which the markers are expressed appear in brown. For the control group, it was evident that the expression of both TNF-α and MMP-9 increased significantly in the stromal layer during the course of the fungal infection. In the plasma treated group, however, the expression of TNF-α was noticeably reduced (with respect to the control group) by day 3. On day 14, measurements showed that TNF-α expression levels had been suppressed to levels characteristic of normal tissue (cf. Fig. 8a). In the case of MMP-9, the expression of this marker by day 14 had also been suppressed significantly (relative to that for the control group) in the plasma-treated group. All of these experiments demonstrate that plasma treatment of corneal tissue inhibits inflammation in *Candida*-infected eyes.

## Discussion

Several significant differences exist between a single, macroscale plasma jet device and microplasma jet arrays with respect to scalability, gas phase chemistry, power consumption, and uniformity of treatment of a surface. Owing to a fill factor of 20–30% over the area encompassed by an array of microplasma columns, the arrays produce a spatially uniform distribution of atomic and molecular species throughout the coverage area, despite the microscale cross-sectional dimensions of each jet. It is these species that are responsible for the beneficial effects on rabbit corneas described in the preceding sections. Previously reported work has demonstrated the efficacy of microplasma jet arrays in disinfection^26^. Although single plasma jets are also capable of generating energetic species and radicals, its limited cross-section restricts the tissue area available for treatment and rastering is often necessary. Furthermore, the power consumption for one conventional jet (1 mm in diameter or more) generally exceeds that for an entire microplasma array. Since the entirety of the electrical power is dissipated in a single jet, the gas temperature in the plasma surpasses that for any of the microscale jets in an array.

Another asset of microplasma jet arrays for medical therapeutics is the level of control with regard to species formation. As one example, the relative intensity of the well-known oxygen atomic line at 777 nm (^5^P–^5^S) can be enhanced while suppressing nitrogen dimer ion (N_2_^+^) emission by simply varying the flow of He through the array or removing the shroud discussed earlier^24^. These and similar effects suggest that the operating parameters of the microplasma arrays can be manipulated so as to favor disinfection or other catalytic reaction processes in a biological system while avoiding the degradation or destruction of tissue. Research on the interaction of microplasma jet arrays with tissue is ongoing, but the control of the plasma chemistry afforded by microplasmas interacting with air or other gaseous environments is clearly an asset for the treatment of a wide range of medical and biological processes. The introduction of a cap (shroud) to confine the plasma chemical region to the vicinity of the tissue allows for the gas phase chemistry to be controlled with exquisite precision and frees the clinician from being restricted to only those radicals and excited species available when the plasma jet interacts with room air. Consequently, the physician is provided with a wider range of treatment options. The ability of the cap to profoundly alter the chemistry occurring in proximity to the tissue is clear from the obvious differences between the two spectra of Fig. 3. Not only does the cap provide a tool for controlling gas chemistry by isolating the plasma jets and their chemical products from room air, but its curvature corresponds to the surface profile of an eye and it is responsible for improving the uniformity in treatment over the desired area of tissue.

A series of experiments has confirmed that the microplasma jet array is better suited to the treatment of keratitis than is a single jet **(**Fig. 4**)**. The corneal epithelium was completely removed during the treatment of rabbit corneas with a single jet, presumably because of the higher power deposition into the plasma (as compared to individual microplasmas). This drawback of the single plasma is problematic because of controlled wound healing, without long-term tissue damage, is necessary for the full recovery of vision following treatment^27^. Even though the application of microplasma jet array did not cause corneal edema, there was a slight reduction of corneal thickness. Preservation of suitable corneal dehydration results from factors including stromal swelling pressure, the barrier function of the epithelium and endothelium, the endothelial pump, evaporation from the corneal surface, and the intraocular pressure^28^. Corneal thickness in microplasma jet could be decreased by the dehydration. However, this cornea dehydration was the transient condition, so the corneal thickness was improved to condition of the normal eye from 1 day after treatment. Furthermore, the increased surface area-to-volume of the microplasma array, relative to a single jet having an equivalent diameter, yields enhanced production of the molecular radicals and excited species essential for promoting the healing of tissue.

It should be mentioned that several polymorpho-nuclear (PMN) cells were detected in the stroma of corneas treated by a single plasma jet. Although Alhabshan *et al*.^27^ concluded that treatment of corneas with atmospheric plasma does not induce corneal inflammation or haze, they did observe a few PMN cells in the anterior stromal tissue. This mild pro-inflammatory response cannot be ignored in terms of medical device development^27^. We conclude that the specific characteristics of plasma jets are critical to their efficacy in medical therapeutics and, specifically, that the microplasma jet arrays yield outcomes in treating keratitis that is preferable to those resulting from a single plasma jet. This result is consistent with the conclusions of our previous study of wound healing with microplasma arrays^20^.

*Candida* keratitis is a potentially blinding disease and can be induced at an epithelial defect by herpes keratitis. Abrasions induced by contaminated contact lenses under certain ocular or systemic conditions can also render the cornea vulnerable to infection^3,19,29,30^. Although the pathogenesis of keratitis by *Candida albicans* is not fully understood, recent studies have suggested a role for inflammatory mediators in the development of corneal ulceration and keratomycosis^31–33^. Infection or inflammation of the cornea also impairs vision because of changes in the refractive index of the tissue and the association of inflammation with edema. The vascularity of the cornea is also critical for maintaining transparency^34^.

When a cornea is infected by *Candida albicans* keratitis, toll-like receptors (TLR) trigger both an immunological reaction and acute inflammation. In response to *Candida albicans*, signaling of the receptor TLR2, in particular, upregulates cytokines, including TNF-α and interleukins which, in turn, increase MMPs and stimulate neovascularization^35,36^. In this study, clinical observations of corneal transparency **(**Fig. 5**)** and the alteration of corneal histology, including corneal edema **(**Fig. 6**)**, angiogenesis **(**Figs 5 and 7**)**, and inflammation **(**Fig. 8**)**, were recorded. Here, PAS staining **(**Fig. 6**)** served as a valuable tool for assessing alterations in the structure of tissue such as basement membranes, capsules, and blood vessels. Stromal edema and damage to the epithelium were observed in *Candida albicans*-infected corneas in Day 1 for both the control and plasma-treated groups. However, *Candida albicans* has been shown previously to be quickly deactivated by low-temperature plasma^22^ and, at Day 14, the stromal swelling was suppressed and the infection had virtually vanished. On the last day of observation (Day 14), a thin layer of epithelium was visible in the plasma-treated corneas.

Clarity of the cornea is also an important parameter for regaining vision, and the microplasma jet array was observed to reduce the corneal opacity and vascularization induced by *Candida albicans*. After 14 days of plasma treatment, the clarity of the corneas had recovered to the level characteristic of the normal corneal tissue. Similarly, immunohistochemistry studies of the angiogenic markers VEGF and CD31, and the inflammatory markers TNF-α and MMP-9, also demonstrated that treatment of rabbit corneas with microplasma jets gradually suppressed the expression of each of these markers in the corneas.

When the microplasma jets interact with room air above the rabbit corneal tissue, we anticipate that particular molecular radicals, commonly known as reactive oxygen species or reactive nitrogen species^22,25^, are primarily responsible for deactivating *Candida albicans* and reversing its effects on a cornea. Specifically, the chemistry of low-temperature plasmas interacting with atmospheric air favors the production of diatomic and triatomic molecules such as NO and O_3_ (respectively) that are capable of deactivating microorganisms and pathogens or serving as dynamic signaling species known to be responsible for therapeutic effects^22,25^. The He microplasma jets of the present study are known to produce large (10^13^–10^14^ cm^−3^) number densities of helium metastable atoms, as well as atomic and dimer He ions. The N_2_^+^ emission observed in Fig. 3(b) from a He microplasma jet interacting with air is the result of charge exchange between He^+^ or He_2_^+^ and a nitrogen molecule in the ground state. The subsequent interaction of this ion with background oxygen or nitrogen produces a variety of species, a few of which are responsible for lipid peroxidation of bacteria membranes^37^.

In summary, the studies described here have confirmed the impact of microplasma treatment of corneas on the edema, angiogenesis, and inflammation produced in rabbit eyes by *Candida albicans*. Future work will focus on experiments designed to standardize the plasma source and the test conditions (including treatment time and the plasma driving voltage and frequency) so as to optimize and standardize the treatment of *Candida albicans* infections of the cornea. Once this therapeutic treatment is thoroughly characterized, it may also prove effective for the plasma treatment of other fungal-related diseases in humans.

## Methods

### Non-thermal atmospheric pressure devices: single plasma jet and eyeball fitted microplasma jet

A brief description of the design and performance of the microplasma jet arrays employed in these experiments has been published previously^24^. A single jet device has an outlet with a 2 mm inner diameter. The high voltage, high-frequency electrode, and ground electrode have widths of 5 mm and separation between two electrodes was 5 mm. The entire structure of microplasma jet array shown in Fig. 1(a) was fabricated by a molding process of a silicon polymer^24^. To control chemical species at the perimeter of microplasma jets we have formed a skirt shaped-circular polyethylene cap at the vicinity of the outlet of microplasma jet array. The cap has a diameter of 3.5 cm and no physical and chemical seal to restrict gas flow were made between the target surface and jet device. The cap that fit close to eyeball prevents the produced plasma from escaping outside eyeball. For all of the experiments reported here, the microchannel diameter (which corresponds to the jet diameter) was 500 µm and pitch (center-to-center spacing) along either the vertical or the horizontal coordinate of the array were fixed at 1 mm (Fig. 1(b)). These correspond to jet packing densities in these arrays of ~130 cm^−2^ in the plane defined by the microchannel exit apertures. The array device fabricated in this paper has 4 × 4 outlets, and spacing between two enclosed discharge electrodes is 1 mm. Both devices operate in helium (He) as a feedstock gas at backing pressures ranging from 780 to 800 Torr. The discharge of both devices was obtained by a 20 kHz sinusoidal voltage waveform excitation, and the root-mean-square (RMS) values of the applying voltage are 0.88–1.06 kV (Fig. 1(c)).

### Wettability test and optical emission spectroscopy

We have tested the surface modification of polypropylene plate with single plasma jet device having an area of 3.8 mm^2^ and array of microplasma jet devices having an area of 12.25 mm^2^ while microplasma jet device has a cap covered its environment over an area of 3.5 cm diameter. To test wettability depending on plasma type, water was dropped on polypropylene plate after plasma treatment for 3 seconds.

The spectral region of 250–850 nm was investigated on CAP jet to detect various oxygen–nitrogen species (RONS)^20^. Briefly, spectra of the emission produced by the microplasma jets expanding into room air were recorded in the UV-vis-NIR region with a collimating lens and a 0.75 m spectrometer equipped with 1800 lines mm^−1^ blazed holographic grating. The estimated resolution of the spectrometer for a slit width of 30 μm is 1.2 × 10^−2^ nm to first order, and because the fluorescence was monitored end-on to the jets, the spectra was considered as being spatially averaged over the entire array.

## Animal Study

Animal care and all experimental procedures were performed in accordance with the Guideline for Animal Experimentation of Inje University Busan Paik Hospital with the approval of the Institutional Animal Care and Use Committee (IACUC No. IJUBPH-2016–007). Forty-eight male New Zealand white rabbits weighing between 2.0 kg and 2.5 kg were obtained from Samtako (Osan, Korea). Systemic anesthesia was induced by intramuscular injection of a mixture of ketamine hydrochloride (30 mg/kg body weight, Huons, Jecheon, Korea) and xylazine hydrochloride (2.5 mg/kg, Bayer Korea Ltd., Seoul, Korea), and topical anesthesia was induced by Alcaine proparacaine eye drops (Alcon Inc., Seoul, Korea).

### Cold atmospheric plasma (CAP) treatment on the normal cornea

In order to investigate possible cellular damage of cornea during any plasma jet treatment, we have performed the treatment with two different types of the jet devices described above in each eye of the same specimen simultaneously (n = 4). In the case of the group of day 0 after plasma treatment, the rabbits’ left and right eyes were treated by plasma for three minutes. In order to avoid drying of the treated cornea specimens, phosphate buffered saline (PBS, 8 g of NaCl, 0.2 g of KCl, 1.44 g of Na_2_HPO_4_, 0.24 g of KH_2_PO_4_, pH 7.4; Gibco, Carlsbad, CA, USA) were added dropwise every 30 s during plasma application. Immediately after plasma treatment, the rabbits were euthanized in a CO_2_ chamber and were removed the eyeball. To observe the alteration of tissue over time after plasma jet treatment, we have performed the treatment with two different type of the jet devices in rabbit’s right eyes only (n = 6, respectively) for 3 minutes. One day and 3 days later, we removed eye ball of rabbit for histological examination. Then, the rabbit’s eyeballs were fixed in 4% formalin to prepare a paraffin block. The paraffin block was sectioned 6 μm and then stained with Hematoxylin and Eosin. To observe the thickness of the rabbit’s cornea following plasma treatment, all tissue slides were observed under a microscope (NanoZoomer 2.0 RS, Hamamatsu photonics, Shizuoka Prefecture, Japan).

### *Candida albicans* infected keratitis on rabbit eyes and CAP treatment

The rabbit keratitis model was developed by applying a 10 mm filter paper soaked in 99% ethanol to rabbits’ right central corneas for one minute (n = 32). Then, the central cornea was gently scraped with No. 15 surgical blade (Ailee Co., Ltd, Busan, Korea) until the stromal layer was exposed. After the removal of the epithelium layer, 50 μL of 10% *Candida albicans* was instilled onto the ocular surface. The left eyes of rabbits were not infected by *Candida* and used as a normal group. At 5 days just before extending corneal neo-vessels to limbus (Supplementary data Fig. S1), the rabbits were randomly divided into two groups: the control group (n = 16), and plasma treatment group (n = 16). The control group contained *Candida*-infected corneas that were not treated with plasma. The plasma treatment group was treated by microplasma jet device after *Candida*-infection. The right eyes were treated by microplasma for three minutes. In order to avoid drying of the treated cornea specimens, PBS was added dropwise every 30 s during plasma application. The rabbits were subdivided into four groups by time point (1, 3, 7 and 14 days after treatments).

### Gross findings: angiogenesis and opacification

The clinical features of the eyes of all rabbits were evaluated on day 1, 3, 7, and 14 after treatment. The eyes were photographed under systemic anesthesia, and corneal angiogenesis and opacification were scored according to the previously described method^38^. Briefly, the extent of corneal angiogenesis was scored from 0 through 3, where 0 = no angiogenesis, 1 = angiogenesis confined to the corneal periphery, 2 = angiogenesis extending up to the pupil margin, and 3 = angiogenesis extending beyond the pupil margin into the central cornea. The severity of corneal opacification was graded from 0 through 3, where 0 = clear cornea with iris details clearly visualized, 1 = partial obscuration of the iris details, 2 = iris details poorly seen with the pupil margin just visible, and 3 = complete obscuration of iris and pupil details.

## Histological Analysis

The rabbits were euthanized at 1, 3, 7 and 14 days after plasma treatment in a CO_2_ chamber. The surgically excised eyeball of the rabbit was fixed in 4% formalin and embedded in paraffin. The tissue was cut with a microtome (RM2245, Leica Biosystems, Nussloch, Germany). The clinical effects of the non-thermal micro jet type of plasma were observed by staining with Periodic Acid-Schiff reagent (PAS) or immunohistochemistry (IHC). PAS staining that is useful to confirm contour of tissue structure can be a good evaluation method of *Candida albicans* corneal infection in animal study^39^. PAS staining of the cornea was performed using a commercial kit (Merck, Darmstadt, Germany) according to the manufacturer’s instructions. Immunohistochemical analysis of cornea was performed according to the previous description^38^. The primary antibodies for Vascular endothelial growth factor (VEGF) and a cluster of differentiation 31 (CD31) were obtained from Bioss Inc. (Woburn, MA, USA) and Abcam Inc. (Cambridge, MA, USA), respectively. The tumor necrosis factor alpha (TNF-α) and matrix metalloproteinase (MMP)-9 were purchased from Lifespan Biosciences Inc. (Seattle, WA, USA). All sections were observed under a microscope (NanoZoomer 2.0 RS, Hamamatsu photonics, Shizuoka Prefecture, Japan).

## Statistical Analysis

All experiments were performed at least three times by conducting each assay in triplicate. Data were analyzed by SPSS version 18.0 for Windows (SPSS, Chicago, IL, USA) and is expressed as the mean ± standard deviation. The statistical analyses were conducted using analysis of variance (ANOVA–Tukey test) between groups, and statistical significance was tested at the *p* < 0.05 levels.

## Electronic supplementary material

Supplementary Information
