# Supplementary material for: Microplasma Jet Arrays as a Therapeutic Choice for Fungal Keratitis
Source: Sci Rep. 2018 Feb 5;8:2422. doi: 10.1038/s41598-018-20854-8 (PMC5799211; doi:10.1038/s41598-018-20854-8)
Supplement: Supplementary file 1 — Supplementary Information [file 41598_2018_20854_MOESM1_ESM.pdf]

Supplementary Information

Microplasma Jet Arrays as a Therapeutic Choice for Fungal Keratitis

Hyun Jung Park[1†], Soon Hee Kim[1†], Hyung Woo Ju[1], Hyesook Lee[2], Yoonjin Lee[2], Sehyun Park[3], Heejun Yang[3], Sung-Jin Park[3], J. Gary Eden[3], Jaewook Yang[2,4*], Chan Hum Park[1,5*]

[1]Nano-Bio Regenerative Medical Institute, Hallym University, Chuncheon, Gangwon, Republic of Korea, [2]T2B infrastructure center for ocular diseases, Inje University Busan Paik Hospital, 75 Bokji-ro, Busanjin-gu, Busan, Republic of Korea, [3]Laboratory for Optical Physics and Engineering, Department of Electrical and Computer Engineering, University of Illinois, Urbana, IL, USA, [4]Department of Ophthalmology, Inje University College of Medicine, 75 Bokji-ro, Busanjin-gu, Busan, Republic of Korea, [5]Department of Otorhinolaryngology–Head and Neck Surgery, Chuncheon Sacred Heart Hospital, School of Medicine, Hallym University, Chuncheon, Gangwon, Republic of Korea

[†]These authors contributed equally to this work. [*]Correspondence and requests for materials should be addressed to C. H. P. (e-mail: hlpch@paran.com), J Yang (e-mail: oculoplasty@gmail.com)

Materials & Methods

Precedent study to decide plasma treatment stage

Animal care and all experimental procedures were performed in accordance with the Guideline for Animal Experimentation of Inje University Busan Paik Hospital with the approval of the Institutional Animal Care and Use Committee (IACUC No. IJUBPH-2016-007). Four male New Zealand white rabbits weighing between 2.0 kg and 2.5 kg were obtained from Samtako (Osan, Korea). Systemic anesthesia was induced by intramuscular injection of a mixture of ketamine hydrochloride (30 mg/kg body weight, Huons, Jecheon, Korea) and xylazine hydrochloride (2.5 mg/kg, Bayer Korea Ltd., Seoul, Korea), and topical anesthesia was induced by Alcaine proparacaine eye drops (Alcon Inc., Seoul, Korea).

The rabbit keratitis model was developed by applying a 10-mm filter paper soaked in 99% ethanol to rabbits' right central corneas for one minute. Then, the central cornea was gently scraped with No. 15 surgical blade (Ailee Co., Ltd, Busan, Korea) until the stromal layer was exposed. After the removal of the epithelium layer, 50 μL of 10% *Candida albicans* was instilled onto the ocular surface. The left eyes of rabbits were not infected by *Candida*. We observed the clinical observation of corneal neovascularization and opacity for 7 days after infection. Images of the eye were obtained with a microscope (SZX7, Olympus, Tokyo, Japan)

Chemical stains for histological analysis to evaluate the safety of microplasma jet array

The rabbits were euthanized at immediately after plasma treatment in a CO_2 chamber. The surgically excised eyeball of the rabbit was fixed in 4% formalin and embedded in paraffin. The tissue was cut to 6 μm with a microtome (RM2245, Leica Biosystems, Nussloch, Germany). Tissue sections stained with hematoxylin/eosin (HE). Images of the sections were photographed with a virtual microscope (NanoZoomer 2.0 RS, Hamamatsu, Japan) under low magnification to evaluate the

safety of plasma treatment to surrounding tissues of cornea. The corneal part in the tissue sections were shown in **Fig. 4A** of the main study.

Results

Precedent study to decide plasma treatment stage

We observed the condition for 7 days after infection of *Candida albicans* in rabbit eyes. As a result of evaluation of corneal neovascularization and opacity, the cornea opacity was indicated from infection of *Candida albicans* and it went from bad to worse on the time dependent manner. At 5 days after infection, the corneal neo-vessels were extended to the limbus. The neovascularization was increased to central cornea on 7 days after infection, and some rabbits were accompanied with a corneal ulcer. The change of clinical observation after 7 days was shown in **Fig. 5** “control” of the main study. Based on this result, we have decided that the stage of pathogenesis following by *Candida albicans* is an appropriate stage for evaluation of efficacy of micro jet type plasma.

Chemical stains for histological analysis to evaluate the safety of microplasma jet array

To evaluate the safety of plasma treatment to surrounding tissues of the cornea, whole eye ball was carried out H&E staining. As shown in Supplementary data (**Fig. S2**), the application of microplasma showed no damages to surrounding tissues such as lens, iris, and retina.

Supplementary Figures

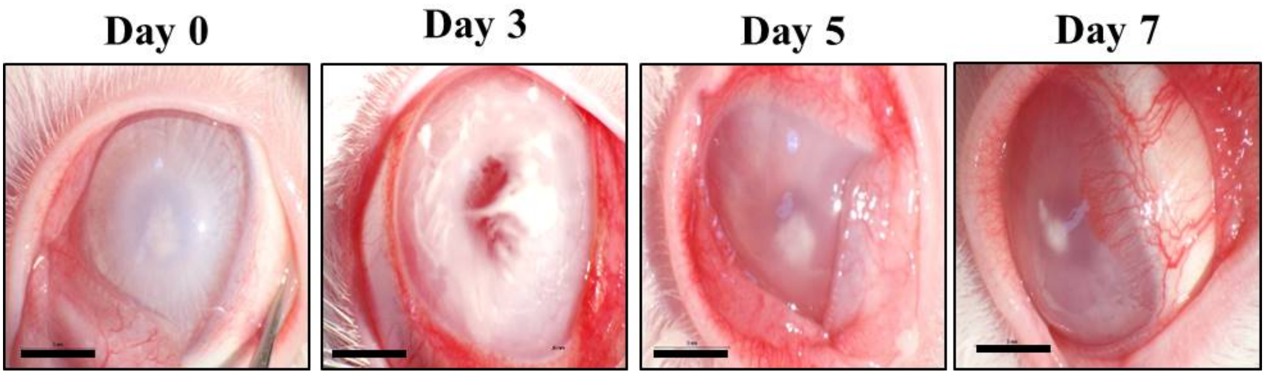

Figure S1. The stage of pathogenesis following by *Candida albicans.* Photographs of rabbits at 0, 3, 5, 7 days after infection of *Candida albicans*. Scale bar, 10 mm.

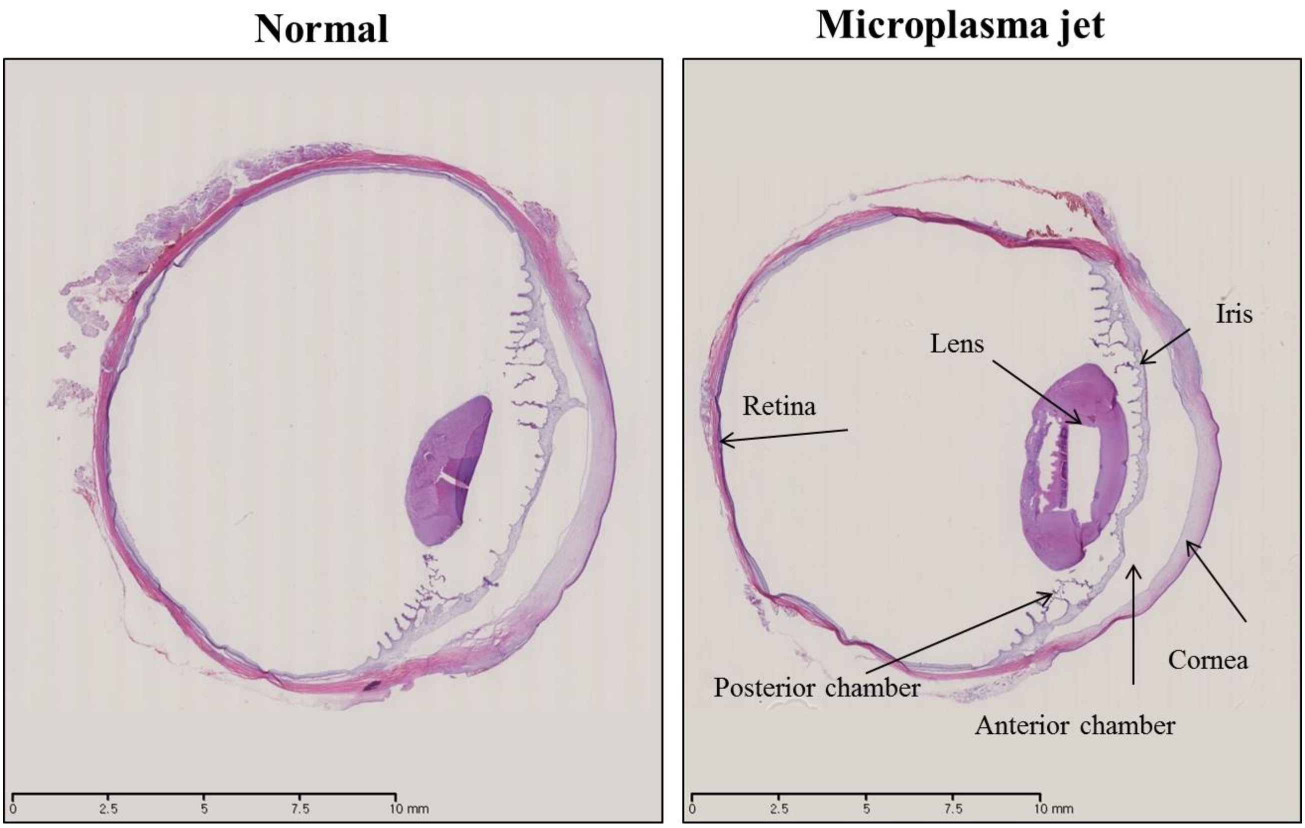

Figure S2. HE staining of whole eye ball to see the safety of microplasma jet array. The application of microplasma showed no damages to the lens, iris, retina and surrounding tissues. Scale bar, 10 mm.
